# Programmed cell death protein‐1 (PD‐1)‐targeted immunotherapy in advanced hepatocellular carcinoma: efficacy and safety data from an international multicentre real‐world cohort

**DOI:** 10.1111/apt.15245

**Published:** 2019-04-12

**Authors:** Bernhard Scheiner, Martha M. Kirstein, Florian Hucke, Fabian Finkelmeier, Kornelius Schulze, Johann von Felden, Sandra Koch, Philipp Schwabl, Jan B. Hinrichs, Fredrik Waneck, Oliver Waidmann, Thomas Reiberger, Christian Müller, Wolfgang Sieghart, Michael Trauner, Arndt Weinmann, Henning Wege, Jörg Trojan, Markus Peck‐Radosavljevic, Arndt Vogel, Matthias Pinter

**Affiliations:** ^1^ Vienna Austria; ^2^ Hannover Germany; ^3^ Klagenfurt Austria; ^4^ Frankfurt/Main Germany; ^5^ Hamburg Germany; ^6^ Mainz Germany

## Abstract

**Background:**

Programmed cell death protein‐1‐targeted immunotherapy has shown promising results in phase II studies of hepatocellular carcinoma.

**Aim:**

To evaluate safety and efficacy of nivolumab and pembrolizumab in an international, multicentre, real‐world cohort of patients with advanced hepatocellular carcinoma.

**Methods:**

Sixty‐five patients treated with nivolumab (n = 34) or pembrolizumab (n = 31) between July 10, 2015 and December 31, 2018 (data cut‐off) across six centres in Austria and Germany were retrospectively analysed.

**Results:**

Child‐Pugh class A/B/C was 32 (49%)/28 (43%)/5 (8%). Immunotherapy was used as systemic first‐/second‐/third‐/fourth‐line treatment in 9 (14%)/27 (42%)/26 (40%)/3 (5%) patients. Fifty‐four patients had at least one follow‐up imaging and were, therefore, available for radiological response assessment. The overall response and disease control rates were 12% and 49% respectively. Of 52 evaluable patients, four (8%) had hyperprogressive disease. Median time to progression was 5.5 (95% CI, 3.5‐7.4) months, median progression‐free survival was 4.6 (95% CI, 3.0‐6.2) months, and median overall survival was 11.0 (95% CI, 8.2‐13.8) months. Most common adverse events were infections (n = 7), rash (n = 6), pruritus (n = 3), fatigue (n = 3), diarrhoea (n = 3) and hepatitis (n = 3). Efficacy and safety results were comparable between Child‐Pugh A and B patients; however, median overall survival (OS) was shorter in Child‐Pugh B patients (16.7 vs 8.6 months; *P* = 0.065). There was no difference in terms of efficacy and adverse events between patients who received immunotherapy as first‐/second‐line and third‐/fourth‐line respectively.

**Conclusions:**

Programmed cell death protein‐1‐targeted immunotherapy with nivolumab or pembrolizumab showed promising efficacy and safety in patients with advanced hepatocellular carcinoma, including subjects with Child‐Pugh stage B and patients with intensive pretreatment.

## 1 INTRODUCTION

Hepatocellular carcinoma (HCC) represents the most common primary liver cancer and usually develops in patients suffering from underlying chronic liver disease.^1^, ^2^, ^3^, ^4^, ^5^ Despite recommendations for surveillance of patients at risk, HCC is often diagnosed at an advanced stage where only systemic treatment can be offered. Many patients develop recurrence or disease progression after initial surgical or loco‐regional treatment and then become candidates for palliative systemic therapy.^1^, ^5^, ^6^ For the last decade, the tyrosine kinase inhibitor sorafenib was the only effective drug available for HCC,^6^ with two randomised controlled phase III trials showing a survival benefit compared to placebo.^7^, ^8^ Only recently, three more tyrosine kinase inhibitors were approved for HCC, lenvatinib in first‐line and regorafenib and cabozantinib in second‐line drug treatment.^9^, ^10^, ^11^ Ramucirumab, a monoclonal antibody against vascular endothelial growth factor receptor (VEGFR)‐2, improved survival in a second‐line phase III study of patients with advanced HCC and elevated alpha‐fetoprotein,^12^ and thus will likely be included in the treatment algorithm shortly.

Immunotherapy with checkpoint blockers demonstrated encouraging efficacy in certain cancer types, particularly in melanoma and lung cancer.^13^ HCC may also be an attractive candidate for immunotherapy, as it represents an immunogenic tumour, but also fosters an immunosuppressive microenvironment (eg, by up‐regulation of immune checkpoint molecules). This may be further supported by the tolerogenic liver milieu and chronic inflammation due to the underlying liver disease.^6^, ^14^, ^15^, ^16^ Notably, overexpression of the checkpoint molecules programmed cell death‐ligand 1 (PD‐L1) and programmed cell death protein‐1 (PD‐1) was associated with tumour aggressiveness and postoperative recurrence in HCC.^17^, ^18^

Nivolumab and pembrolizumab, two monoclonal antibodies against PD‐1, have shown promising efficacy and safety results in noncomparative, open‐label phase II studies of advanced HCC,^19^, ^20^ and the United States Food and Drug Administration (FDA) already granted accelerated conditional approval to both agents for sorafenib‐experienced patients with HCC. Both nivolumab and pembrolizumab are currently being investigated in ongoing phase III trials.

In the present study, we aimed to analyse the safety and efficacy of anti‐PD‐1 targeted therapy with nivolumab or pembrolizumab in an international, multicentre, real‐life cohort of patients with advanced HCC. In contrast to the phase II studies of nivolumab and pembrolizumab,^19^, ^20^ our cohort also includes patients with more advanced liver cirrhosis (Child‐Pugh B/C) as well as patients who received immunotherapy as third or even fourth line of systemic therapy. Thus, this cohort reflects the treatment reality in advanced HCC outside of clinical trial programs.

## 2 PATIENTS AND METHODS

### 2.1 Study design and patients

This was a retrospective study of patients treated with nivolumab or pembrolizumab across six centres in Austria and Germany. Patients with histologically or radiologically confirmed HCC^1^ who received PD‐1‐targeted immunotherapy with nivolumab or pembrolizumab were eligible. All data, including patient history, laboratory results and radiological information were collected retrospectively. The retrospective analysis was approved by local Ethics Committees.

### 2.2 Dosing of nivolumab and pembrolizumab

Nivolumab was administered at 1‐3 mg/kg body weight or at a fixed dose of 240 mg every 2 weeks intravenously. Pembrolizumab was given at 2 mg/kg body weight or at a fixed dose of 200 mg every 3 weeks intravenously. Dose delays were made based on toxicity.

### 2.3 Assessments

Radiological response was recorded by computed tomography (CT) or magnetic resonance imaging (MRI) at baseline, 6‐12 weeks after treatment initiation, and about every 3 months thereafter. Tumour response was assessed according to the modified Response Evaluation Criteria in Solid Tumours (mRECIST).^21^ Patients with progressive disease of target lesions (increase of at least 20%) at the first radiological evaluation were assessed for hyperprogressive disease. Hyperprogression was defined as a progressive disease (RECIST version 1.1^22^) on the first radiological evaluation during immunotherapy with a delta tumour growth rate of > 50%, corresponding to an absolute increase in tumour growth rate exceeding 50% per month.^23^ Tumour growth rate was calculated as described previously,^23^ and delta tumour growth rate (tumour growth rate during immunotherapy minus tumour growth rate before immunotherapy) was then used to assess the association of immunotherapy with tumour growth. Tumour growth rate was only quantified for target lesions.^23^ To calculate tumour growth rate before and during immunotherapy, images of CT/MRI scans were required from three different time points: (a) before baseline (while patient was receiving prior therapy/no therapy), (b) at baseline (before initiation of immunotherapy), (c) at first evaluation during immunotherapy. Hence, hyperprogressive disease could only be calculated in patients of whom radiographic images were available from all three above mentioned time points.

Side effects were recorded at every visit and graded according to the Common Terminology Criteria for Adverse Events (CTCAE) version 4.

### 2.4 Statistics

Data on baseline characteristics, radiological tumour response and side effects were summarised using descriptive statistics. Chi square test or Fisher's exact test were used to compare nominal data. We avoided statistical comparison between nivolumab‐ and pembrolizumab‐treated patients as this would be unreliable due to the retrospective study design. Median duration of treatment was defined as time from the date of the first administration until the date of last infusion. Patients who were still receiving immunotherapy at data cut‐off were censored. Patients who had at least one follow‐up imaging assessment were evaluable for radiological response and time to progression. Time to progression (TTP) was defined as the time from the date of first checkpoint inhibitor administration until the date of first radiologically confirmed tumour progression. Data from patients who died without radiologically confirmed tumour progression were censored at the date of last radiological assessment. Progression‐free survival (PFS) was defined as the time from the date of first checkpoint inhibitor administration until radiological disease progression or death, whatever came first. Patients who were still alive and without radiologically confirmed progression were censored at the date of last contact or data cut‐off. Overall survival (OS) was defined as the time from start of immunotherapy until the date of death. Patients who were still alive were censored at the date of last contact or data cut‐off. Survival curves were calculated using the Kaplan‐Meier method and compared by means of the log rank test. Statistical analyses were performed using IBM SPSS Statistics version 25 (SPSS Inc., Chicago, IL). A *P* < 0.05 was considered significant.

## 3 RESULTS

### 3.1 Patients

**Figure 1:**
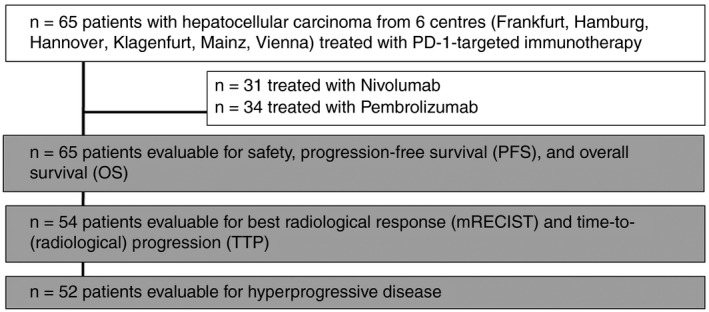
Patient flow chart

Sixty‐five patients in whom PD‐1 targeted immunotherapy was initiated between July 10, 2015 and April 27, 2018 were included. The date of data cut‐off was December 31, 2018. Thirty‐four subjects received nivolumab and 31 patients were treated with pembrolizumab (Figure 1). Main baseline characteristics are shown in Table 1. Immunotherapy was used as systemic first‐, second‐, third‐, or fourth‐line treatment in 9 (14%), 27 (42%), 26 (40%) and 3 (5%) patients respectively. Agents used for prior systemic therapy are shown in Table S1. Fifty‐one (79%) patients had advanced stage HCC and a significant number of patients had Child‐Pugh stage B/C (n = 33; 51%).

**Table 1:** Baseline characteristics

	Nivolumab, n = 34	Pembrolizumab, n = 31	All patients, n = 65
Age (y), mean ± SD	64.0 ± 10.6	66.5 ± 11.7	65.2 ± 11.1
Sex			
Male	24 (71%)	25 (81%)	49 (75%)
Female	10 (29%)	6 (19%)	16 (25%)
Aetiology			
Alcohol	5 (15%)	14 (45%)	19 (29%)
Hepatitis C	8 (24%)	2 (6%)	10 (15%)
Hepatitis B	5 (15%)	3 (10%)	8 (12%)
NAFLD	8 (24%)	3 (10%)	11 (7%)
Other	8 (24%)	9 (29%)	17 (26%)
Child‐Pugh stage			
A	17 (50%)	15 (48%)	32 (49%)
B	14 (41%)	14 (45%)	28 (43%)
C	3 (9%)	2 (6%)	5 (8%)
ECOG PS			
0	16 (47%)	16 (52%)	32 (49%)
≥1	18 (53%)	15 (48%)	33 (51%)
Prior treatment			
Surgery	8 (24%)	7 (23%)	15 (23%)
Ablation	5 (15%)	4 (13%)	9 (14%)
Loco‐regional (TACE, SIRT, radiation)	12 (35%)	18 (58%)	30 (46%)
Systemic	28 (82%)	28 (90%)	56 (86%)
Previous sorafenib	28 (82%)	28 (90%)	56 (86%)
Previous regorafenib	10 (29%)	15 (48%)	25 (38%)
Immunotherapy as systemic			
First‐line	6 (18%)	3 (10%)	9 (14%)
Second‐line	17 (50%)	10 (32%)	27 (42%)
Third‐line	9 (27%)	17 (55%)	26 (40%)
Fourth‐line	2 (6%)	1 (3%)	3 (5%)
Macrovascular invasion	13 (38%)	11 (36%)	24 (37%)
Extrahepatic metastasis	21 (62%)	14 (45%)	35 (54%)
BCLC stage			
B	2 (6%)	6 (19%)	8 (12%)
C	28 (82%)	23 (74%)	51 (79%)
D	4 (12%)	2 (6%)	6 (9%)
Alpha‐Fetoprotein			
<400 (IU/ml)	20 (59%)	16 (52%)	36 (55%)
≥400 (IU/ml)	13 (38%)	15 (48%)	28 (43%)

BCLC, Barcelona‐Clinic Liver Cancer; ECOG PS, Eastern Cooperative Oncology Group Performance Status; NAFLD, non‐alcoholic fatty liver disease; SIRT, selective internal radiotherapy; TACE, transarterial chemoembolisation.

Median duration of follow‐up was 11.2 (95% CI, 9.9‐12.6) months. Median time of treatment was 3.1 (95% CI, 2.5‐3.6) months for nivolumab and 2.8 (95% CI, 0‐5.7) months for pembrolizumab. At data cut‐off, 2 (6%) and 9 (29%) patients were still on treatment with nivolumab and pembrolizumab respectively. Immunotherapy was discontinued mainly due to radiological or clinical disease progression (nivolumab and pembrolizumab, n = 23 (68%) and n = 14 (45%)) and adverse events (nivolumab and pembrolizumab, n = 6 (18%) and n = 1 (3%)). After discontinuation of immunotherapy, 21 (32%) patients received an alternative treatment. The following therapies were used: regorafenib (n = 5), lenvatinib (n = 5), ramucirumab (n = 4), radiation (n = 4), sorafenib (n = 2), cabozantinib (n = 2), capecitabine (n = 2), gemcitabine plus cisplatin (n = 1), microwave ablation (n = 1) and SIRT (n = 1).

### 3.2 Efficacy

Fifty‐four patients had at least one follow‐up imaging and were therefore available for radiological tumour response assessment (nivolumab, n = 30; pembrolizumab, n = 24). Of the 11 subjects not available for response assessment, 9 patients died before the first radiological evaluation and 2 patients were lost to follow‐up. In the nivolumab group, no patient had complete response (CR) and 5 (15%) participants achieved partial response (PR), resulting in an overall response rate (ORR) of 15%. Ten (29%) patients showed stable disease (SD) and 15 (44%) subjects had progressive disease at first radiological evaluation. The disease control rate (DCR) was 44%. In the pembrolizumab‐treated patients, 0 and 3 (10%) participants achieved complete response and partial response, respectively. Fourteen (45%) patients had stable disease and 7 (23%) individuals showed progressive disease. The overall response rate and disease control rate were 10% and 55% respectively. The overall response rate and disease control rate for the whole cohort were 12% and 49% respectively (Table 2). Of 54 patients with at least one follow‐up imaging, 52 patients were evaluable for hyperprogression (nivolumab, n = 28; pembrolizumab, n = 24), of which 4 (8%) subjects were classified as having hyperprogressive disease (nivolumab, n = 2 (7%); pembrolizumab, n = 2 (8%)).

**Table 2:** Radiological response according to mRECIST and survival

	Nivolumab	Pembrolizumab	All patients
Best response			
CR	0	0	0
PR	5 (15%)	3 (10%)	8 (12%)
SD	10 (29%)	14 (45%)	24 (37%)
PD	15 (44%)	7 (23%)	22 (34%)
Not evaluable	4 (12%)	7 (23%)	11 (17%)
ORR (CR+PR)	15%	10%	12%
DCR (CR+PR+SD)	44%	55%	49%
PFS, median (95% CI)	4.3 (2.0‐6.7) mo	5.6 (1.1‐10.1) mo	4.6 (3.0‐6.2) mo
TTP, median (95% CI)	4.6 (1.9‐7.4) mo	6.4 (3.4‐9.5) mo	5.5 (3.5‐7.4) mo
OS, median (95% CI)	9.0 (5.5‐12.5) mo	11.0 (7.4‐14.5) mo	11.0 (8.2‐13.8) mo
1‐year survival rate	38%	44%	42%

CR, complete response; DCR, disease control rate; mRECIST, modified Response Evaluation Criteria in Solid Tumours; ORR, overall response rate; OS, overall survival; PD, progressive disease; PFS, progression‐free survival; PR, partial response; SD, stable disease; TTP, time to progression.

**Figure 2:**
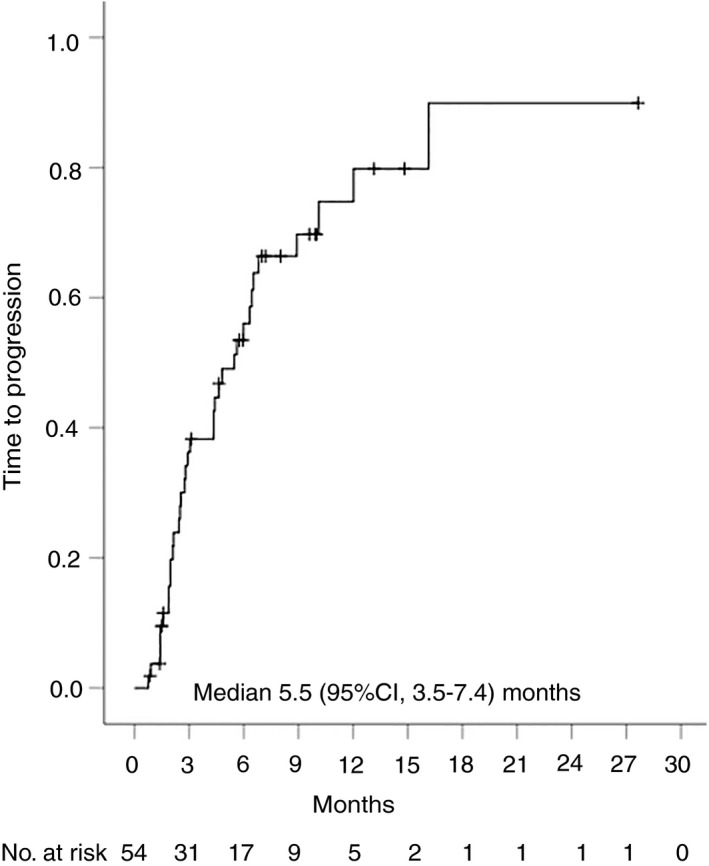
Kaplan‐Meier curve showing time to progression for the whole cohort of patients treated with programmed cell death protein‐1 (PD‐1)‐targeted immunotherapy

**Figure 3:**
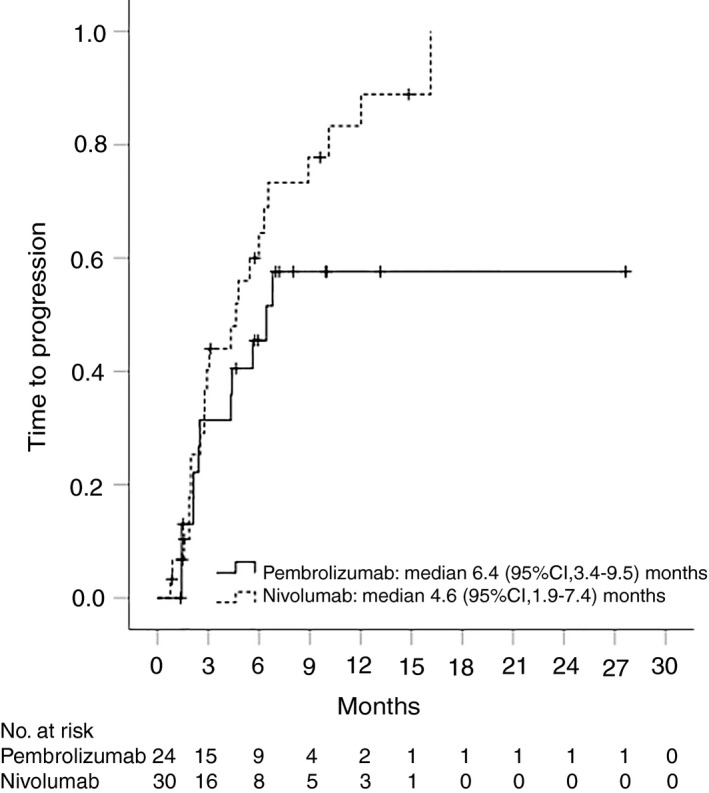
Kaplan‐Meier curves showing time to progression for nivolumab‐ and pembrolizumab‐treated patients

**Figure 4:**
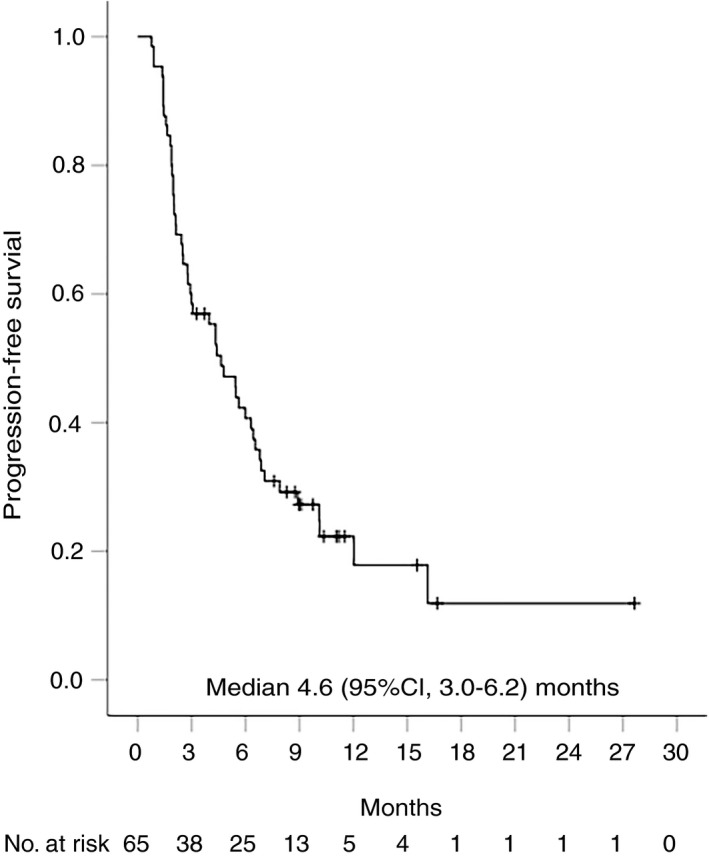
Kaplan‐Meier curve showing progression‐free survival for the whole cohort of patients treated with programmed cell death protein‐1 (PD‐1)‐targeted immunotherapy

**Figure 5:**
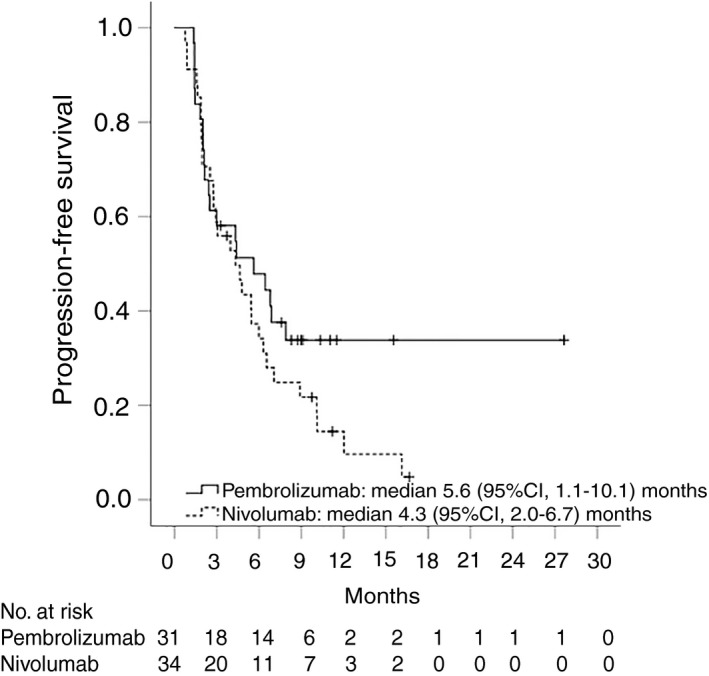
Kaplan‐Meier curves showing progression‐free survival for nivolumab‐ and pembrolizumab‐treated patients

**Figure 6:**
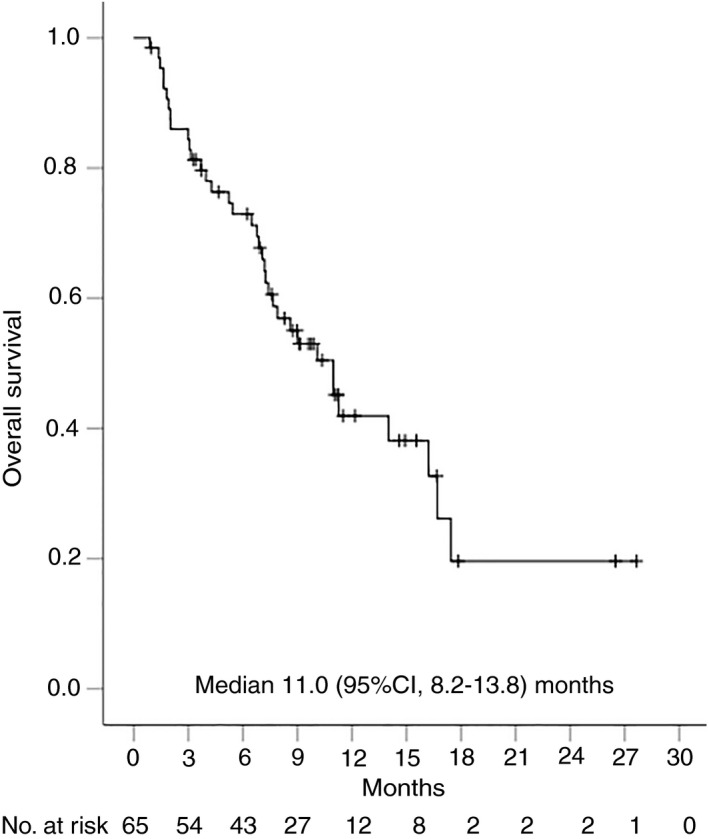
Kaplan‐Meier curve showing overall survival for the whole cohort of patients treated with programmed cell death protein‐1 (PD‐1)‐targeted immunotherapy

**Figure 7:**
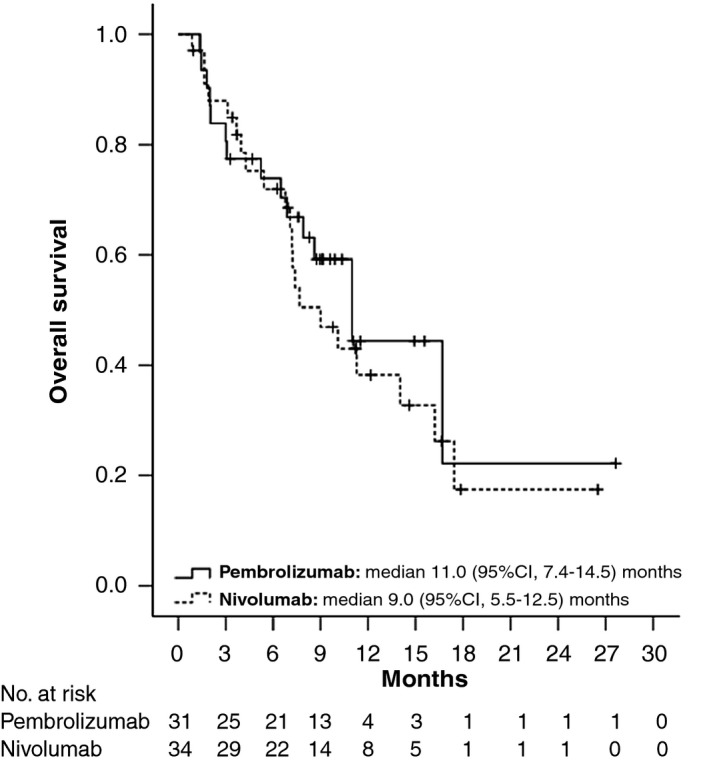
Kaplan‐Meier curves showing overall survival for nivolumab‐ and pembrolizumab‐treated patients

Overall, 35 (54%) patients had radiological disease progression and 36 (55%) participants died during follow‐up. Median time to progression was 5.5 (95% CI, 3.5‐7.4) months for the whole cohort (Figure 2), 4.6 (95% CI, 1.9‐7.4) months for nivolumab and 6.4 (95% CI, 3.4‐9.5) months for pembrolizumab (Figure 3, Table 2). Progression‐free survival was 4.6 (95% CI, 3.0‐6.2) months for the whole group (Figure 4), and 4.3 (95% CI, 2.0‐6.7) months and 5.6 (95% CI, 1.1‐10.1) months for nivolumab and pembrolizumab respectively (Figure 5, Table 2). Median overall survival was 11.0 (95% CI, 8.2‐13.8) months for the whole cohort (Figure 6), 9.0 (95% CI, 5.5‐12.5) months for nivolumab, and 11.0 (95% CI, 7.4‐14.5) months for pembrolizumab (Figure 7, Table 2).

**Figure 8:**
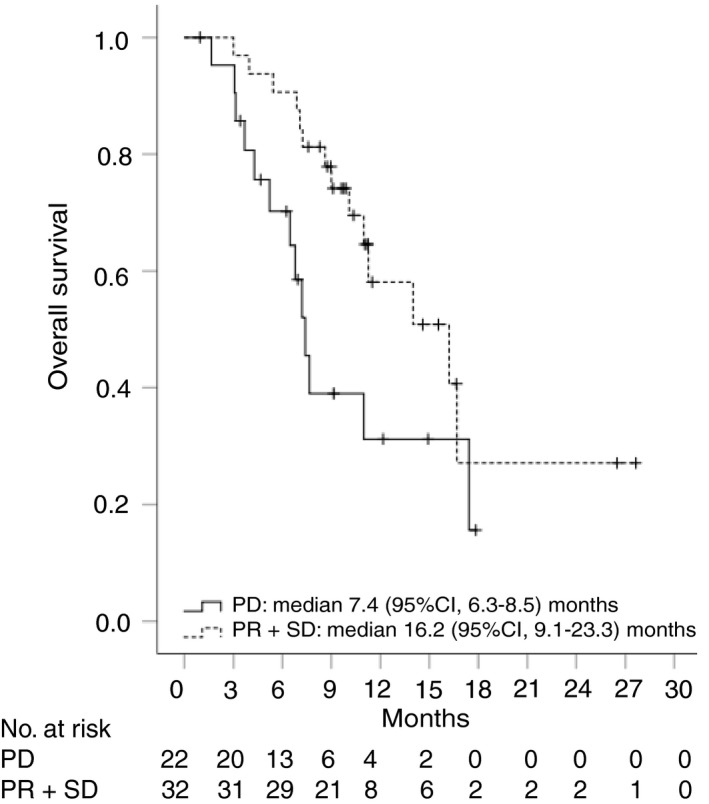
Kaplan‐Meier curves showing overall survival for patients treated with PD‐1‐targeted immunotherapy according to radiological tumour response (partial response (PR)/stable disease (SD) vs progressive disease (PD))

Median OS for patients with partial response or stable disease was 16.2 (95% CI, 9.1‐23.3) months and was significantly longer compared to that of patients with progressive disease, which was 7.4 (95% CI, 6.3‐8.5; *P* = 0.039) months (Figure 8).

### 3.3 Safety

Twenty‐five (39%) patients experienced at least one adverse event (AE). Most common adverse events were infections (n = 7; 11%), rash (n = 6; 9%), pruritus (n = 3; 5%), fatigue (n = 3; 5%), diarrhoea (n = 3; 5%) and hepatitis (n = 3; 5%). All cases of hepatitis were treated with corticosteroids. Eleven (17%) patients developed adverse events of higher grade (grade ≥ 3). Adverse events observed in nivolumab‐ and pembrolizumab‐treated patients are shown in Table 3. One patient died due to an infection; a relationship to pembrolizumab is unlikely but cannot be excluded. A dose delay due to adverse events was required in 6 (18%) patients treated with nivolumab and in 11 (36%) participants receiving pembrolizumab. Steroids or immunosuppressive drugs were used to treat an adverse event in 5 (15%) nivolumab‐ and 5 (16%) pembrolizumab‐treated subjects.

**Table 3:** Adverse events

	Nivolumab, n = 34		Pembrolizumab, n = 31		All patients, n = 65	
	Any grade	Grade ≥ 3	Any grade	Grade ≥ 3	Any grade	Grade ≥ 3
Infection	1 (3%)	1 (3%)	6 (19%)	1 (3%)	7 (11%)	2 (3%)
Rash	2 (6%)	—	4 (13%)	—	6 (9%)	—
Pruritus	—	—	3 (10%)	—	3 (5%)	—
Fatigue	—	—	3 (10%)	—	3 (5%)	—
Hepatitis	2 (6%)	2 (6%)	1 (3%)	1 (3%)	3 (5%)	3 (5%)
Diarrhoea	—	—	3 (10%)	—	3 (5%)	—
Myalgia/Myositis	1 (3%)	1 (3%)	1 (3%)	—	2 (3%)	1 (2%)
Amylase/Lipase increase	—	—	2 (6%)	1 (3%)	2 (3%)	1 (2%)
Vasculitis	—	—	2 (6%)	2 (6%)	2 (3%)	2 (3%)
Mucositis	—	—	2 (6%)	—	2 (3%)	—
Paraesthesia	—	—	1 (3%)	1 (3%)	1 (2%)	1 (2%)
Arthritis	—	—	1 (3%)	—	1 (2%)	—
Thyreoiditis	1 (3%)	—	—	—	1 (2%)	—
Bronchiolitis	—	—	1 (3%)	—	1 (2%)	—
Dyspnoea	—	—	1 (3%)	—	1 (2%)	—
Pain	—	—	1 (3%)	—	1 (2%)	—
Nausea	—	—	1 (3%)	—	1 (2%)	—
Renal	—	—	1 (3%)	—	1 (2%)	—
Allergic reaction	—	—	1 (3%)	—	1 (2%)	—
Gastric ulcer	—	—	1 (3%)	—	1 (2%)	—
Variceal bleeding	1 (3%)	1 (3%)	—	—	1 (2%)	1 (2%)

### 3.4 Efficacy and safety according to Child‐Pugh stage

As the number of patients with Child‐Pugh stage C was too low (n = 5) for meaningful analysis, we excluded these patients and only compared the efficacy and safety of immunotherapy in patients with Child‐Pugh stage A and B. Overall response rate and disease control rate for Child‐Pugh A vs B was 9% vs 14% (*P* = 0.438) and 56% vs 46% (*P* = 0.947) respectively. Median time to progression was 4.8 (95% CI, 2.4‐7.2) months for Child‐Pugh A and 5.5 (95% CI, 1.5‐9.4) months for Child‐Pugh B (*P* = 0.511). Similarly, there was no difference in median progression‐free survival, which was 4.4 (95% CI, 1.2‐7.7) months for Child‐Pugh A and 4.6 (95% CI, 1.4‐7.9) months for Child‐Pugh B (*P* = 0.333). Median overall survival was 16.7 (95% CI, 8.2‐25.2) months for Child‐Pugh stage A and 8.6 (95% CI, 4.8‐12.4) months for Child‐Pugh B (*P* = 0.065) (Table S2).

In terms of safety, there was no difference regarding the number of patients who developed any grade (Child‐Pugh A vs B, n = 10 (31%) vs n = 12 (43%); *P* = 0.352) or high‐grade (Child‐Pugh A vs B, n = 5 (16%) vs n = 5 (18%); *P* = 1.000) adverse events. Adverse events according to Child‐Pugh stage are shown in Table S3.

### 3.5 Efficacy and safety according to systemic line of immunotherapy

As the number of patients who received immunotherapy as first (n = 9) or fourth (n = 3) line of systemic treatment was low, we grouped patients who received immunotherapy as first‐ or second‐line (group 1) and those in whom immunotherapy was used as third‐ or fourth‐line of systemic treatment (group 2).

Overall response rate and disease control rate for group 1 vs 2 was 11% vs 14% (*P* = 1.000) and 50% vs 48% (*P* = 0.901) respectively. Median time to progression was 5.6 (95% CI, 3.0‐8.3) months for group 1 and 4.8 (95% CI, 1.5‐8.1) months for group 2 (*P* = 0.452). Median progression‐free survival was 4.3 (95% CI, 0.9‐7.8) months for group 1 and 4.8 (95% CI, 1.8‐7.8) months for group 2 (*P* = 0.652). Median overall survival was 11.0 (95% CI, 5.7‐16.3) months for group 1 and 10.1 (95% CI, 7.4‐12.7) months for group 2 (*P* = 0.893) (Table S4).

In terms of safety, the number of patients who developed any grade (Group 1 vs 2, n = 12 (33%) vs n = 13 (45%); *P* = 0.344) or high‐grade (Group 1 vs 2, n = 6 (17%) vs n = 5 (17%); *P* = 1.000) adverse events was similar between group 1 and 2.

## 4 DISCUSSION

We demonstrate that PD‐1‐targeted immunotherapy with nivolumab or pembrolizumab showed promising efficacy and mild toxicity in a real‐world cohort of patients with advanced stage HCC. Efficacy and safety results were comparable between Child‐Pugh A and B patients, even though median overall survival was shorter in Child‐Pugh B patients (16.7 vs 8.6 months). Overall survival of patients with stable disease or partial response was significantly longer than that of subjects with progressive disease (16.2 vs 7.4 months).

Two phase II studies tested nivolumab and pembrolizumab in patients with intermediate‐advanced stage HCC. The CheckMate 040 study, an open‐label, noncomparative, phase I/II trial, tested nivolumab in sorafenib‐naïve (n = 80) and ‐experienced (n = 182) patients with HCC and Child‐Pugh class A.^19^ Nivolumab was well‐tolerated with fatigue, pruritus, rash and diarrhoea being the most common adverse events. Overall response rate was 23% in sorafenib‐naïve and 19% in sorafenib‐pretreated patients according to RECISTv1.1 assessed by investigators. Responses were durable, independent of PD‐L1 expression, and translated into an encouraging survival with a median overall survival of 28.6 months in sorafenib‐naïve and around 15 months in sorafenib‐experienced patients. The few patients with a complete or partial response had an excellent outcome with 18‐ and 45‐months survival rates of 100% and about 90% respectively.^19^, ^24^, ^25^ Based on these promising data, nivolumab was conditionally approved for HCC previously treated with sorafenib in the United States in 2017. Recently published data from the KEYNOTE‐224 trial,^20^ a nonrandomised, open‐label phase II study investigating pembrolizumb in sorafenib‐pretreated patients (n = 104) with Child‐Pugh stage A showed similar results with an overall response rate of 17%, a median progression‐free survival of 4.9 months, and a median overall survival of 12.9 months. Again, fatigue, pruritus, diarrhoea and rash were the most frequent side effects.^20^ Pembrolizumab also received FDA approval in the United States recently. Nivolumab as the first‐line treatment and pembrolizumab in second‐line are currently investigated in ongoing phase III trials of advanced HCC (nivolumab: NCT02576509; pembrolizumab: NCT02702401, NCT03062358).

Monoclonal antibodies are not metabolized by the liver but eliminated predominantly via uptake and catabolism by the reticuloendothelial system and target tissue.^26^ This could make the pharmacokinetic profile of immune checkpoint inhibitors more predictable even in patients with advanced liver cirrhosis.^15^ However, the CheckMate 040^19^ and the KEYNOTE‐224^20^ study only included Child‐Pugh A patients, a common practice in HCC trials in order to minimise the confounding effect of death from liver cirrhosis on overall outcome.^27^ In our study, a significant proportion of patients had Child‐Pugh stage B or C (51%) though. The number of patients with any grade and high‐grade adverse events was similar between Child‐Pugh class A and B suggesting that immunotherapy can be administered safely even in patients with more advanced liver function impairment. Even though efficacy in terms of overall response rate, time to progression and progression‐free survival was similar between Child‐Pugh A and B patients, OS was shorter in the Child‐Pugh B group, and prognosis of patients with advanced HCC and Child‐Pugh stage B is often limited.^28^ Thus, the decision to use systemic treatment in decompensated patients should be evaluated carefully on a case‐by‐case basis, taking into account other comorbidities and—most importantly—the potential for recompensation.

In contrast to the phase II trials of nivolumab^19^ and pembrolizumab^20^ that used immunotherapy as first‐ or second‐line treatment, we administered checkpoint blockers as third or even fourth line in nearly half of patients (45%). Despite the intensive pretreatment, immunotherapy led to a disease stabilization in about half of the patients. However, in contrast to the CheckMate 040^19^ and the KEYNOTE‐22^20^ study, which used RECIST v1.1 to assess their primary endpoint, none of our patients had complete response, even though we used mRECIST criteria, which have a higher sensitivity to capture response to treatment compared to conventional RECIST.^1^

Efficacy and safety was similar between patients who received immunotherapy as first‐/second‐line treatment compared to those in whom immunotherapy was used in third‐/fourth‐line. Most agents used prior to immunotherapy in our cohort (eg, sorafenib, regorafenib) are known for their anti‐angiogenic effects.^29^, ^30^ This is of particular interest as hypoxia, often induced by anti‐angiogenic agents, promotes an immunosuppressive tumour microenvironment, inter alia by an upregulation of immune checkpoint molecules.^31^, ^32^ Indeed, sorafenib intensified tumour hypoxia and increased tumoural PD‐L1 expression in experimental models of HCC.^33^, ^34^ Thus, immunotherapy may be particularly attractive following or combined with anti‐vascular endothelial growth factor (VEGF)‐targeted therapies. In line, preliminary data of pilot studies testing the combination of lenvatinib plus pembrolizumab (n = 26) and bevacizumab combined with atezolizumab (n = 68) showed encouraging response rates of 42% and 34% respectively.^35^, ^36^

Hyperprogressive disease—an increased tumour growth rate during treatment—is a new pattern of progression that was recently reported for patients treated with PD‐1‐/PD‐L1‐targeted immunotherapy.^23^, ^37^, ^38^ Four (8%) patients in our cohort had hyperprogression during immunotherapy. This is in line with a previous study that reported hyperprogressive disease in 9% of patients with advanced cancers,^37^ but lower compared to recurrent and/or metastatic head and neck cancer (29%)^38^ and advanced non‐small‐cell lung cancer (13.8%) treated with PD‐1/PD‐L1 blockers.^23^ Notably, hyperprogressive disease was defined differently in these studies as there is currently no consensus on the optimal definition.^23^, ^37^, ^38^ The underlying mechanisms for hyperprogressive disease are unknown, but it was hypothesised that major immune reactions, promotion of tumour cell proliferation, immune compensatory mechanisms and prior irradiation may play a role in hyperprogression with PD‐1‐/PD‐L1‐targeted therapy.^37^, ^38^

Despite the retrospective nature and the lack of a control group, the strength of our study is the provision of unique real‐world data on a patient cohort usually excluded from clinical trials (ie, Child‐Pugh B/C, multiple lines of systemic pretreatment). These data represent important new information on subgroups of patients frequently found in everyday clinical practice and tumour board discussions. Additionally, to our knowledge, this is the first study that evaluated hyperprogression with PD‐1 blockers in HCC.

In conclusion, PD‐1‐targeted immunotherapy with nivolumab or pembrolizumab was safe in patients with advanced HCC including patients with Child‐Pugh class B. Immunotherapy was associated with a good survival in those who achieved disease stabilization, while prognosis of patients with progressive disease remained rather poor. This highlights the need for biomarkers to select those patients most likely to benefit from treatment. The combination of immunotherapy and targeted therapies may have the potential to further improve the outcome. A closer radiological follow‐up within the first weeks after initiation of immunotherapy may be considered in order to detect those patients with early progression or hyperprogressive disease. Phase III trials testing nivolumab and pembrolizumab in the first‐ and second‐line setting are ongoing and their results are eagerly awaited.

## FUNDING INFORMATION

None.

## AUTHORSHIP

*Guarantor of the article*: Matthias Pinter.

*Author contributions*: All authors contributed either to research design (B.S.; M.M.K; and M.P.), and/or the data acquisition (B.S., M.M.K., F.H., F.F., K.S. J.v.F., S.K., P.S. J.B.H., O.W., T.R., A.W., H.W., J.T., A.V., M.P.), analysis (B.S. and M.P.), or interpretation (all authors) of data. B.S., M.M.K. and M.P. drafted the manuscript, which was critically revised by all other authors. All authors approved the final version of the manuscript.

## APPENDIX 1 The authors’ complete affiliations

### 1.1

Bernhard Scheiner, Division of Gastroenterology and Hepatology, Department of Internal Medicine III, Liver Cancer (HCC) Study Group Vienna, Vienna Hepatic Hemodynamic Laboratory, Medical University of Vienna, Vienna, Austria; Martha M. Kirstein, Department of Gastroenterology, Hepatology and Endocrinology, Hannover Medical School, Hannover, Germany; Florian Hucke, Department of Internal Medicine and Gastroenterology (IMuG), Hepatology, Endocrinology, Rheumatology and Nephrology including Centralized Emergency Department (ZAE), Klinikum Klagenfurt am Wörthersee, Klagenfurt, Austria; Fabian Finkelmeier, Department of Gastroenterology, Hepatology and Endocrinology, University Hospital Frankfurt, Frankfurt/Main, Germany; Kornelius Schulze, 1. Department of Internal Medicine, Gastroenterology & Hepatology, University Medical Center Hamburg‐Eppendorf, Hamburg, Germany; Johann von Felden, 1. Department of Internal Medicine, Gastroenterology & Hepatology, University Medical Center Hamburg‐Eppendorf, Hamburg, Germany; Sandra Koch, Department of Internal Medicine, University Medical Center of the Johannes Gutenberg University Mainz, Mainz, Germany; Philipp Schwabl, Division of Gastroenterology and Hepatology, Department of Internal Medicine III, Vienna Hepatic Hemodynamic Laboratory, Medical University of Vienna, Vienna, Austria; Jan B. Hinrichs, Institute for Radiology, Hannover Medical School, Hannover, Germany; Fredrik Waneck, Division of Cardiovascular & Interventional Radiology, Department of Biomedical Imaging and Image‐guided Therapy, Medical University of Vienna, Vienna, Austria; Oliver Waidmann, Department of Gastroenterology, Hepatology and Endocrinology, University Hospital Frankfurt, Frankfurt/Main, Germany; Thomas Reiberger, Division of Gastroenterology and Hepatology, Department of Internal Medicine III, Vienna Hepatic Hemodynamic Laboratory, Medical University of Vienna, Vienna, Austria; Christian Müller, Division of Gastroenterology and Hepatology, Department of Internal Medicine III, Liver Cancer (HCC) Study Group Vienna, Medical University of Vienna, Vienna, Austria; Wolfgang Sieghart, Division of Gastroenterology and Hepatology, Department of Internal Medicine III, Liver Cancer (HCC) Study Group Vienna, Medical University of Vienna, Vienna, Austria; Michael Trauner, Division of Gastroenterology and Hepatology, Department of Internal Medicine III, Medical University of Vienna, Vienna, Austria; Arndt Weinmann, Department of Internal Medicine, University Medical Center of the Johannes Gutenberg University Mainz, Mainz, Germany; Henning Wege, 1. Department of Internal Medicine, Gastroenterology & Hepatology, University Medical Center Hamburg‐Eppendorf, Hamburg, Germany; Jörg Trojan, Department of Gastroenterology, Hepatology and Endocrinology, University Hospital Frankfurt, Frankfurt/Main, Germany; Markus Peck‐Radosavljevic, Department of Internal Medicine and Gastroenterology (IMuG), Hepatology, Endocrinology, Rheumatology and Nephrology including Centralized Emergency Department (ZAE), Klinikum Klagenfurt am Wörthersee, Klagenfurt, Austria; Arndt Vogel, Department of Gastroenterology, Hepatology and Endocrinology, Hannover Medical School, Hannover, Germany; Matthias Pinter, Division of Gastroenterology and Hepatology, Department of Internal Medicine III, Liver Cancer (HCC) Study Group Vienna, Medical University of Vienna, Vienna, Austria.

## References

[1] Galle PR , Forner A , Llovet JM , et al. EASL Clinical Practice Guidelines: management of hepatocellular carcinoma. J Hepatol. 2018;69:182‐236. 29628281 10.1016/j.jhep.2018.03.019

[2] Hucke F , Sieghart W , Schoniger‐Hekele M , Peck‐Radosavljevic M , Muller C . Clinical characteristics of patients with hepatocellular carcinoma in Austria ‐ is there a need for a structured screening program? Wien Klin Wochenschr. 2011;123:542‐551. 21800047 10.1007/s00508-011-0033-9

[3] Pinter M , Hucke F , Zielonke N , Trauner M , Sieghart W , Peck‐Radosavljevic M . Epidemiological trends of hepatocellular carcinoma in Austria. Dig Dis. 2014;32:664‐669. 25376282 10.1159/000367983

[4] Pinter M , Trauner M , Peck‐Radosavljevic M , Sieghart W . Cancer and liver cirrhosis: implications on prognosis and management. ESMO Open. 2016;1:e000042. 27843598 10.1136/esmoopen-2016-000042 PMC5070280

[5] Vogel A , Cervantes A , Chau I , et al. Hepatocellular carcinoma: ESMO Clinical Practice Guidelines for diagnosis, treatment and follow‐up. Ann Oncol. 2018;29:iv238‐iv255. 30285213 10.1093/annonc/mdy308

[6] Pinter M , Peck‐Radosavljevic M . Review article: systemic treatment of hepatocellular carcinoma. Aliment Pharmacol Ther. 2018;48:598‐609. 30039640 10.1111/apt.14913 PMC6120553

[7] Cheng AL , Kang YK , Chen Z , et al. Efficacy and safety of sorafenib in patients in the Asia‐Pacific region with advanced hepatocellular carcinoma: a phase III randomised, double‐blind, placebo‐controlled trial. Lancet Oncol. 2009;10:25‐34. 19095497 10.1016/S1470-2045(08)70285-7

[8] Llovet JM , Ricci S , Mazzaferro V , et al. Sorafenib in advanced hepatocellular carcinoma. N Engl J Med. 2008;359:378‐390. 18650514 10.1056/NEJMoa0708857

[9] Bruix J , Qin S , Merle P , et al. Regorafenib for patients with hepatocellular carcinoma who progressed on sorafenib treatment (RESORCE): a randomised, double‐blind, placebo‐controlled, phase 3 trial. Lancet. 2017;389:56‐66. 27932229 10.1016/S0140-6736(16)32453-9

[10] Kudo M , Finn RS , Qin S , et al. Lenvatinib versus sorafenib in first‐line treatment of patients with unresectable hepatocellular carcinoma: a randomised phase 3 non‐inferiority trial. Lancet. 2018;391:1163‐1173. 29433850 10.1016/S0140-6736(18)30207-1

[11] Abou‐Alfa GK , Meyer T , Cheng AL , et al. Cabozantinib in patients with advanced and progressing hepatocellular carcinoma. N Engl J Med. 2018;379:54‐63. 29972759 10.1056/NEJMoa1717002 PMC7523244

[12] Zhu AX , Kang YK , Yen CJ , et al. Ramucirumab after sorafenib in patients with advanced hepatocellular carcinoma and increased alpha‐fetoprotein concentrations (REACH‐2): a randomised, double‐blind, placebo‐controlled, phase 3 trial. Lancet Oncol. 2019;20:282‐296. 30665869 10.1016/S1470-2045(18)30937-9

[13] Smyth MJ , Ngiow SF , Ribas A , Teng MW . Combination cancer immunotherapies tailored to the tumour microenvironment. Nat Rev Clin Oncol. 2016;13:143‐158. 26598942 10.1038/nrclinonc.2015.209

[14] Greten TF , Wang XW , Korangy F . Current concepts of immune based treatments for patients with HCC: from basic science to novel treatment approaches. Gut. 2015;64:842‐848. 25666193 10.1136/gutjnl-2014-307990 PMC6311419

[15] Hato T , Goyal L , Greten TF , Duda DG , Zhu AX . Immune checkpoint blockade in hepatocellular carcinoma: current progress and future directions. Hepatology. 2014;60:1776‐1782. 24912948 10.1002/hep.27246 PMC4211962

[16] Finkelmeier F , Waidmann O , Trojan J . Nivolumab for the treatment of hepatocellular carcinoma. Expert Rev Anticancer Ther. 2018;18:1169‐1175. 30304963 10.1080/14737140.2018.1535315

[17] Gao Q , Wang XY , Qiu SJ , et al. Overexpression of PD‐L1 significantly associates with tumor aggressiveness and postoperative recurrence in human hepatocellular carcinoma. Clin Cancer Res. 2009;15:971‐979. 19188168 10.1158/1078-0432.CCR-08-1608

[18] Shi F , Shi M , Zeng Z , et al. PD‐1 and PD‐L1 upregulation promotes CD8(+) T‐cell apoptosis and postoperative recurrence in hepatocellular carcinoma patients. Int J Cancer. 2011;128:887‐896. 20473887 10.1002/ijc.25397

[19] El‐Khoueiry AB , Sangro B , Yau T , et al. Nivolumab in patients with advanced hepatocellular carcinoma (CheckMate 040): an open‐label, non‐comparative, phase 1/2 dose escalation and expansion trial. Lancet. 2017;389:2492‐2502. 28434648 10.1016/S0140-6736(17)31046-2 PMC7539326

[20] Zhu AX , Finn RS , Edeline J , et al. Pembrolizumab in patients with advanced hepatocellular carcinoma previously treated with sorafenib (KEYNOTE‐224): a non‐randomised, open‐label phase 2 trial. Lancet Oncol. 2018;19:940‐952. 29875066 10.1016/S1470-2045(18)30351-6

[21] Lencioni R , Llovet JM . Modified RECIST (mRECIST) assessment for hepatocellular carcinoma. Semin Liver Dis. 2010;30:52‐60. 20175033 10.1055/s-0030-1247132 PMC12268942

[22] Eisenhauer EA , Therasse P , Bogaerts J , et al. New response evaluation criteria in solid tumours: revised RECIST guideline (version 1.1). Eur J Cancer. 2009;45:228‐247. 19097774 10.1016/j.ejca.2008.10.026

[23] Ferrara R , Mezquita L , Texier M , et al. Hyperprogressive disease in patients with advanced non‐small cell lung cancer treated with PD‐1/PD‐L1 inhibitors or with single‐agent chemotherapy. JAMA Oncol. 2018;4:1543‐1552. 30193240 10.1001/jamaoncol.2018.3676 PMC6248085

[24] Crocenzi TS , El‐Khoueiry AB , Yau TC , et al. Nivolumab (nivo) in sorafenib (sor)‐naive and ‐experienced pts with advanced hepatocellular carcinoma (HCC): CheckMate 040 study. J Clin Oncol. 2017;35(suppl; abstr 4013):4013.

[25] El‐Khoueiry AB , Melero I , Yau TC , et al. Impact of antitumor activity on survival outcomes, and nonconventional benefit, with nivolumab (NIVO) in patients with advanced hepatocellular carcinoma (aHCC): subanalyses of CheckMate‐040. J Clin Oncol. 2018;36(suppl 4S; abstr 475):475.

[26] Newsome BW , Ernstoff MS . The clinical pharmacology of therapeutic monoclonal antibodies in the treatment of malignancy; have the magic bullets arrived? Br J Clin Pharmacol. 2008;66:6‐19. 18503606 10.1111/j.1365-2125.2008.03187.x PMC2485255

[27] Llovet JM , Di Bisceglie AM , Bruix J , et al. Design and endpoints of clinical trials in hepatocellular carcinoma. J Natl Cancer Inst. 2008;100:698‐711. 18477802 10.1093/jnci/djn134

[28] Marrero JA , Kudo M , Venook AP , et al. Observational registry of sorafenib use in clinical practice across Child‐Pugh subgroups: the GIDEON study. J Hepatol. 2016;65:1140‐1147. 27469901 10.1016/j.jhep.2016.07.020

[29] Sun MY , Wu SX , Zhou XB , Gu JM , Hu XR . Comparison of the crystal structures of the potent anticancer and anti‐angiogenic agent regorafenib and its monohydrate. Acta Crystallogr C Struct Chem. 2016;72:291‐296. 27045179 10.1107/S2053229616003727

[30] Wilhelm SM , Adnane L , Newell P , Villanueva A , Llovet JM , Lynch M . Preclinical overview of sorafenib, a multikinase inhibitor that targets both Raf and VEGF and PDGF receptor tyrosine kinase signaling. Mol Cancer Ther. 2008;7:3129‐3140. 18852116 10.1158/1535-7163.MCT-08-0013 PMC12261297

[31] Jain RK . Antiangiogenesis strategies revisited: from starving tumors to alleviating hypoxia. Cancer Cell. 2014;26:605‐622. 25517747 10.1016/j.ccell.2014.10.006 PMC4269830

[32] Pinter M , Jain RK . Targeting the renin‐angiotensin system to improve cancer treatment: implications for immunotherapy. Sci Transl Med. 2017;9:eaan5616. 28978752 10.1126/scitranslmed.aan5616 PMC5928511

[33] Chen Y , Huang Y , Reiberger T , et al. Differential effects of sorafenib on liver versus tumor fibrosis mediated by stromal‐derived factor 1 alpha/C‐X‐C receptor type 4 axis and myeloid differentiation antigen‐positive myeloid cell infiltration in mice. Hepatology. 2014;59:1435‐1447. 24242874 10.1002/hep.26790 PMC3966948

[34] Chen Y , Ramjiawan RR , Reiberger T , et al. CXCR4 inhibition in tumor microenvironment facilitates anti‐programmed death receptor‐1 immunotherapy in sorafenib‐treated hepatocellular carcinoma in mice. Hepatology. 2015;61:1591‐1602. 25529917 10.1002/hep.27665 PMC4406806

[35] Ikeda M , Sung MW , Kudo M , et al. A phase 1b trial of lenvatinib (LEN) plus pembrolizumab (PEM) in patients (pts) with unresectable hepatocellular carcinoma (uHCC). J Clin Oncol. 2018;36 (suppl; abstr 4076):4076.

[36] Pishvaian MJ , Lee MS , Ryoo B‐Y , et al. Updated safety and clinical activity results from a phase Ib study of atezolizumab + bevacizumab in hepatocellular carcinoma (HCC). Ann Oncol. 2018;29 (suppl_8):abstr LBA26.

[37] Champiat S , Dercle L , Ammari S , et al. Hyperprogressive disease is a new pattern of progression in cancer patients treated by anti‐PD‐1/PD‐L1. Clin Cancer Res. 2017;23:1920‐1928. 27827313 10.1158/1078-0432.CCR-16-1741

[38] Saada‐Bouzid E , Defaucheux C , Karabajakian A , et al. Hyperprogression during anti‐PD‐1/PD‐L1 therapy in patients with recurrent and/or metastatic head and neck squamous cell carcinoma. Ann Oncol. 2017;28:1605‐1611. 28419181 10.1093/annonc/mdx178

