# Supplementary material for: Programmed cell death protein‐1 (PD‐1)‐targeted immunotherapy in advanced hepatocellular carcinoma: efficacy and safety data from an international multicentre real‐world cohort
Source: Aliment Pharmacol Ther. 2019 Apr 12;49(10):1323–33. doi: 10.1111/apt.15245 (PMC6593858; doi:10.1111/apt.15245)
Supplement: Supplementary file 1 [file APT-49-1323-s001.docx]

**Supplemental tables**

| **Supplemental table 1** Agents used prior to immunotherapy in first-, second-, and third-line | | | |
| --- | --- | --- | --- |
| **Agent** | **First-line, n=56 (100%)** | **Second-line, n=29 (100%)** | **Third-line, n=3 (100%)** |
| Sorafenib | 54 (96%) | 2 (7%) | − |
| FOLFIRINOX | 1 (2%) | − | − |
| GEMCIS | 1 (2%) | − | − |
| Regorafenib | − | 22 (76%) | 3 (100%) |
| Tivantinib/Placebo | − | 2 (7%) | − |
| Ramucirumab | − | 1 (3%) | − |
| Doxorubicin | − | 2 (7%) | − |
| Abbreviations: FOLFIRINOX, folinic acid, fluorouracil, irinotecan, oxaliplatin; GEMCIS, gemcitabine, cisplatin. | | | |

| **Supplemental table 2** Radiological response and survival according to Child-Pugh stage | | | |
| --- | --- | --- | --- |
|  | **Child-Pugh A** | **Child-Pugh B** | **P-value** |
| **ORR (CR+PR)** | 9% | 14% | 0.438 (Fisher’s Exact) |
| **DCR (CR+PR+SD)** | 56% | 46% | 0.947 (Chi Square) |
| **PFS, median (95%CI)** | 4.4 (1.2-7.7) months | 4.6 (1.4-7.9) months | 0.333 (log rank) |
| **TTP, median (95%CI)** | 4.8 (2.4-7.2) months | 5.5 (1.5-9.4) months | 0.511 (log rank) |
| **OS, median (95%CI)** | 16.7 (8.2-25.2) | 8.6 (4.8-12.4) | 0.065 (log rank) |
| Abbreviations: DCR, disease control rate; mRECIST, modified Response Evaluation Criteria in Solid Tumors; ORR, overall response rate; OS, overall survival; PFS, progression-free survival; TTP, time to progression | | | |

| **Supplemental table 3** Adverse events according to Child-Pugh stage | | | | |
| --- | --- | --- | --- | --- |
|  | **Child-Pugh A (n=32)** | | **Child-Pugh B (n=28)** | |
|  | Any grade | Grade ≥3 | Any grade | Grade ≥3 |
| Infection | 3 (9%) | 1 (3%) | 3 (11%) | 1 (4%) |
| Rash | − | − | 5 (18%) | − |
| Pruritus | 1 (3%) | − | 2 (7%) | − |
| Fatigue | 1 (3%) | − | 2 (7%) | − |
| Hepatitis | 2 (6%) | 2 (6%) | 1 (4%) | 1 (4%) |
| Diarrhea | 1 (3%) | − | 2 (7%) | − |
| Myalgia/Myositis | 2 (6%) | 1 (3%) | − | − |
| Amylase/lipase increase | 1 (3%) | 1 (3%) | 1 (4%) | − |
| Vasculitis | − | − | 2 (7%) | 2 (7%) |
| Mucositis | 1 (3%) | − | 1(4%) | − |
| Paresthesia | − | − | − | − |
| Arthritis | 1 (3%) | − | − | − |
| Thyroiditis | 1 (3%) | − | − | − |
| Bronchiolitis | − | − | 1 (4%) | − |
| Dyspnea | − | − | 1 (4%) | − |
| Pain | − | − | 1 (4%) | − |
| Nausea | 1 (3%) | − | − | − |
| Renal | − | − | 1 (4%) | − |
| Allergic reaction | 1 (3%) | − | − | − |
| Gastric ulcer | − | − | 1 (4%) | − |
| Variceal bleeding | − | − | 1 (4%) | 1 (4%) |

| **Supplemental table 4** Radiological response and survival according to line of immunotherapy | | | |
| --- | --- | --- | --- |
|  | **Immunotherapy as first-/second-line** | **Immunotherapy as third-/fourth-line** | **P-value** |
| **ORR (CR+PR)** | 11% | 14% | 1.000 (Fisher’s Exact) |
| **DCR (CR+PR+SD)** | 50% | 48% | 0.901 (Chi Square) |
| **PFS, median (95%CI)** | 4.3 (0.9-7.8) months | 4.8 (1.8-7.8) months | 0.652 (log rank) |
| **TTP, median (95%CI)** | 5.6 (3.0-8.3) months | 4.8 (1.5-8.1) months | 0.452 (log rank) |
| **OS, median (95%CI)** | 11.0 (5.7-16.3) months | 10.1 (7.4-12.7) months | 0.893 (log rank) |
| Abbreviations: DCR, disease control rate; mRECIST, modified Response Evaluation Criteria in Solid Tumors; ORR, overall response rate; OS, overall survival; PFS, progression-free survival; TTP, time to progression | | | |
